# Supplementary material for: Assessment of Phenotype Relevant Amino Acid Residues in TEM-β-Lactamases by Mathematical Modelling and Experimental Approval
Source: Microorganisms. 2021 Aug 13;9(8):1726. doi: 10.3390/microorganisms9081726 (PMC8399295; doi:10.3390/microorganisms9081726)
Supplement: Supplementary file 1 [file microorganisms-09-01726-s001.zip › Supplementary Tables and Figures.pdf]

microorganisms

MDPI

Table S1. Primers used in this study.

Name	Sequence 5′→ 3′
**mutagenesis**	
AT-L40V-GTG-for	GATGCTGAAGATCAGGTGGGTGCACGAGTGG
AT-L40V-GTG-rev	CCACTCGTGCACCCACCTGATCTTCAGCATC
SN-M/I69-ATT-for	GAACGTTTTCCAATGATTAGCACTTTTAAAGTTCTG
SN-M/I69-ATT-rev	CAGAACTTTAAAAGTGCTAATCATTGGAAAACGTTC
SN-M/L69-CTG-for	GAACGTTTTCCAATGCTGAGCACTTTTAAAGTTCTG
SN-M/L69-CTG-rev	CAGAACTTTAAAAGTGCTCAGCATTGGAAAACGTTC
SN-M/V69-GTG-for	GAACGTTTTCCAATGGTGAGCACTTTTAAAGTTCTG
SN-M/V69-GTG-rev	CAGAACTTTAAAAGTGCTCACCATTGGAAAACGTTC
AT-N100S-AGT-for	TCAACCAAGTCACTCTGAGAATAGTGTATGCGGCGA
AT-N100S-AGT-rev	TCGCCGCATACACTATTCTCAGAGTGACTTGGTTGA
SN-E/K104-AAG-for	CAGAATGACTTGGTTAAGTACTCACCAGTCACAG
SN-E/K104-AAG-rev	CTGTGACTGGTGAGTACTTAACCAAGTCATTCTG
AT-S/G130-GGT-for	GTGCTGCCATAACCATGGGTGATAACACTGCTGCC
AT-S/G130-GGT-rev	GGCAGCAGTGTTATCACCCATGGTTATGGCAGCAC
S130T-AGT:ACT-FOR	CAGTGCTGCCATAACCATGACTGATAACACTGC
S130T-AGT:ACT-REV	GTTGGCCGCAGTGTTATCAGTCATGGTTATGG
AT-R/C164-TGT-for	GTAACTCGCCTTGATTGTTGGGAACCGGAGC
AT-R/C164-TGT-rev	GCTCCGGTTCCCAACAATCAAGGCGAGTTAC
AT-R/H164-CAT-for	GTAACTCGCCTTGATCATTGGGAACCGGAGCTG
AT-R/H164-CAT-rev	CAGCTCCGGTTCCCAATGATCAAGGCGAGTTAC
AT-R/S164-AGT-for	GTAACTCGCCTTGATAGTTGGGAACCGGAGC
AT-R/S164-AGT-rev	GCTCCGGTTCCCAACTATCAAGGCGAGTTAC
AT-W/C165-TGT-FOR	CTCGCCTTGATCGTTGTGAACCGGAGCTGAATG
AT-W/C165-TGT-REV	CATTCAGCTCCGGTTCACAACGATCAAGGCGAG
AT-W/G165-GGG-FOR	CATGTAACTCGCCTTGATCGTGGGGAACCGGAG
AT-W/G165-GGG-REV	CTCCGGTTCCCCACGATCAAGGCGAGTTACATG
AT-W/L165-TTG-for	GTAACTCGCCTTGATCGTTTGGAACCGGAGC

AT-W/L165-TTG-rev	GCTCCGGTTCCAAACGATCAAGGCGAGTTAC
AT-W/R165-CGG-for	CATGTAACTCGCCTTGATCGTCGGGAACCGGAG
AT-W/R165-CGG-rev	CTCCGGTTCCCGACGATCAAGGCGAGTTACATG
D179E-GAC:GAG-for	TACCAAACGACGAGCGTGAGACCACGATGC
D179E-GAC:GAG-rev	GCATCGTGGTCTCACGCTCGTCGTTTGGTA
M182T-ATG:ACG-for	GAGCGTGACACCACGACGCCTGCAGCAAT
M182T-ATG:ACG-rev	ATTGCTGCAGGCGTCGTGGTGTCACGCTC
L221M-CTG:ATG-for	GATAAAGTTGCAGGACCACTTATGCGCTCGGCC
L221M-CTG:ATG-rev	GGCCGAGCGCATAAGTGGTCCTGCAACTTTATC
AT-G/S238-AGT-for	GATAAATCTGGAGCCAGTGAGCGTGGGTCTC
AT-G/S238-AGT-rev	GAGACCCACGCTCACTGGCTCCAGATTTATC
AT-G/R-238-CGT-for	GATAAATCTGGAGCCCGTGAGCGTGGGTCTC
AT-G/R-238-CGT-rev	GAGACCCACGCTCACGGGCTCCAGATTTATC
AT-E/G-240-GGG-for	CTGGAGCCGGTGGGCGTGGGTCTCG
AT-E/G-240-GGG-rev	CGAGACCCACGCCCACCGGCTCCAG
AT-E/K240-AAG-for	ATCTGGAGCCGGTAAGCGTGGGTCTC
AT-E/K240-AAG-rev	GAGACCCACGCTTACCGGCTCCAGAT
AT-E/R-240-AGG-FOR	TGCTGATAAATCTGGAGCCGGTAGGCGTGGGTCTCG
AT-E/R-240-AGG-REV	CGAGACCCACGCCTACCGGCTCCAGATTTATCAGCA
AT-E/V-240-GTG-for	CTGGAGCCGGTGTGCGTGGGTCTCG
AT-E/V-240-GTG-rev	CGAGACCCACGCACACCGGCTCCAG
AT-R/C-244-TGC-for	CCGGTGAGCGTGGGTCTTGCGGTATCA
AT-R/C-244-TGC-rev	TGATACCGCAAGACCCACGCTCACCGG
AT-R/G-241-GGC-FOR	CCGGTGAGCGTGGGTCTGGCGGTATCA
AT-R/G-241-GGC-REV	TGATACCGCCAGACCCACGCTCACCGG
AT-R/H-244-CAC-for	GTGAGCGTGGGTCTCACGGTATCATTGCAGC
AT-R/H-244-CAC-rev	GCTGCAATGATACCGTGAGACCCACGCTCAC
AT-R/L-244-CTC-FOR	GTGAGCGTGGGTCTCTCGGTATCATTGCAGC
AT-R/L-244-CTC-REV	GCTGCAATGATACCGAGAGACCCACGCTCAC
SN-R/S244-AGC-for	GAGCGTGGGTCTAGCGGTATCATTGCAG
SN-R/S244-AGC-rev	CTGCAATGATACCGCTAGACCCACGCTC
AT-T265M-ATG-FOR	CCGTATCGTAGTTATCTACATGACGGGGAGTCAG
AT-T265M-ATG-REV	CTGACTCCCCGTCATGTAGATAACTACGATACGG
AT-R/A275-GCA-FOR	TCAGGCAACTATGGATGAAGCAAATAGACAGATCGCTGAG
AT-R/A275-GCA-REV	CTCAGCGATCTGTCTATTTGCTTCATCCATAGTTGCCTGA
AT-R/L275-CTA-for	CAGGCAACTATGGATGAACTAAATAGACAGATCGCTGAG
AT-R/L275-CTA-rev	CTCAGCGATCTGTCTATTTAGTTCATCCATAGTTGCCTG
AT-R/Q275-CAA-for	CAGGCAACTATGGATGAACAAAATAGACAGATCGCTGAG
AT-R/Q275-CAA-rev	CTCAGCGATCTGTCTATTTTGTTCATCCATAGTTGCCTG
SN-N/D276-GAT-for	CTATGGATGAACGAGATAGACAGATCGCTG
SN-N/D276-GAT-rev	CAGCGATCTGTCTATCTCGTTCATCCATAG
I279T-ATC:ACC-for	ACTATGGATGAACGAAATAGACAGACCGCTGAGATAGGT
I279T-ATC:ACC-rev	ACCTATCTCAGCGGTCTGTCTATTTCGTTCATCCATAGT
SM_TEM_PaPb_for	TGACATTAACCTATAAAAATAAGCGTATCACGAGGCCCTTTC
SM_TEM_PaPb_rev	GAAAGGGCCTCGTGATACGCTTATTTTTATAGGTTAATGTCA
SM_TEM_P4_for	ATTTACCAGGGTTATTGTATCATGAGCGGATACATATTTGAATGTA
SM_TEM_P4_rev	TACATTCAAATATGTATCCGCTCATGATACAATAACCCTGG-TAAAT

SM_TEM_P5_for	CTCATGAGCGGATACATATTTCAATGTATTTAGAAAAA-TAAACAAATAGGGG
SM_TEM_P5_rev	CCCCTATTTGTTTATTTTTCTAAATACATTGAAA-TATGTATCCGCTCATGAG
**sequencing**	
M13 Forward	GTAAAACGACGGCCAG
M13 Reverse	CAGGAAACAGCTATGAC
**amplification for cloning**	
TEM1-P-XhoI-for	GGAAATTGCTCGAGAGCTCAGTATTGC
TEM1-XbaI-rev	GAGTAAACTTGGTCTAGAAGTTACCAATGC
**expression analysis**	
SN-tem1-stop-T-rev	GGTCTGACAGTTACCAATGCTTAATC
SN-tem1-P-for	GCTCAGTATTGCCCGCTCCA

Table S2. Antibiotics and their solvents used in this study

**Name**	**Solvent**	**Provider**
Amoxicillin	DMSO	TCI Deutschland GmbH, Eschborn, Germany
Ampicillin Sodium Salt	water	Carl Roth GmbH & Co.KG., Karlsruhe, Ger-many
Avibactam Sodium	DMSO	Advanced ChemBlocks Inc., Burlingame, USA
Aztreonam	satured $NaHCO_3$	Sigma Aldrich Chemie GmbH, Taufkirchen, Germany
Cefepime hydrochloride	phosphate buffer 0.1 M pH 6	TCI Deutschland GmbH, Eschborn, Germany
Cefotaxime sodium salt	water	TCI Deutschland GmbH, Eschborn, Germany
Ceftazidime hydrate	0.9 % NaCl	Sigma Aldrich Chemie GmbH, Taufkirchen, Germany
Ceftriaxone disodium salt, hemiheptahydrate	water	TCI Deutschland GmbH, Eschborn, Germany
Ceftobiprole	DMSO	Basilea Pharmaceutica AG
Piperacillin sodium salt	water	Sigma Aldrich Chemie GmbH, Taufkirchen, Germany
Potassium clavulanate	water	Sigma Aldrich Chemie GmbH, Taufkirchen, Germany
Sulbactam	phosphate buffer 0,1 M pH 7.2	Sigma Aldrich Chemie GmbH, Taufkirchen, Germany
Tazobactam	DMSO	Sigma Aldrich Chemie GmbH, Taufkirchen, Germany
Tetracycline hydrochloride	water	AppliChem GmbH, Darmstadt, Germany

Table S3. Strains and plasmids used or generated in this study

Strain Name	Plasmid	Description	Reference
/	pCR™-Blunt II-TOPO	*lacZα*, *ccdB*, Kan^R, Zeo^R, pUC origin, M13R, SP6 promoter, T7 promoter, M13F	Invitrogen
/	pBT	*lacZα*, *tetL*, *ColE1*, M13R, T3, T7 promoter, M13F	Agilent Technologies
/	Topo-P3-TEM-1	pCR™-Blunt II-TOPO with P3-TEM-1	this study
/	pBT-P3-TEM-1	pBT with P3-TEM-1 cloned between *Xho*I and *Xba*I	this study
XL1-blue	none	*recA1*, *endA1* , *gyrA96*, *thi*-1, *hsdR17*, *supE44*, *relA1 lac*[F; *proAB*, *lacIqZΔM15*, Tn10 (Tet^R)]	Agilent Technologies
Mach1™-$T1^R$:	none	F-; *φ80(lacZ)ΔM15*, *ΔlacX74*, *hsdR(rk-, mk+)*, *ΔrecA1398*, *endA1*, *tonA*	Invitrogen
SM-1	Topo-P3-TEM-1	derivative of XL1-blue	this study
SM-2	pBT-P3-TEM-1	derivative of XL1-blue	this study
JH-02	Topo-P3-TEM-L40V	derivative of XL1-blue	this study
HZ-02	Topo-P3-TEM-M69I	derivative of XL1-blue	this study
HZ-03	Topo-P3-TEM-M69L	derivative of XL1-blue	this study
HZ-04	Topo-P3-TEM-M69V	derivative of XL1-blue	this study
HZ-05	Topo-P3-TEM-E104K	derivative of XL1-blue	this study
JH-04	Topo-P3-TEM-S130G	derivative of XL1-blue	this study
HD-02	Topo-P3-TEM-S130T	derivative of XL1-blue	this study
HZ-08	Topo-P3-TEM-R164C	derivative of XL1-blue	this study
HZ-07	Topo-P3-TEM-R164H	derivative of XL1-blue	this study
HZ-06	Topo-P3-TEM-R164S	derivative of XL1-blue	this study
HD-04	Topo-P3-TEM-W165C	derivative of XL1-blue	this study
HD-03	Topo-P3-TEM-W165G	derivative of XL1-blue	this study
JH-05	Topo-P3-TEM-W165L	derivative of XL1-blue	this study
JH-06	Topo-P3-TEM-W165R	derivative of XL1-blue	this study
HD-14	Topo-P3-TEM-M182T	derivative of XL1-blue	this study
JH-07	Topo-P3-TEM-G238R	derivative of XL1-blue	this study
HZ-09	Topo-P3-TEM-G238S	derivative of XL1-blue	this study
HZ-11	Topo-P3-TEM-E240G	derivative of XL1-blue	this study
HD-05	Topo-P3-TEM-E240K	derivative of XL1-blue	this study
HD-06	Topo-P3-TEM-E240R	derivative of XL1-blue	this study
HD-07	Topo-P3-TEM-E240V	derivative of XL1-blue	this study
JH-08	Topo-P3-TEM-R244C	derivative of XL1-blue	this study
HD-08	Topo-P3-TEM-R244G	derivative of XL1-blue	this study
JH-09	Topo-P3-TEM-R244H	derivative of XL1-blue	this study
HD-09	Topo-P3-TEM-R244L	derivative of XL1-blue	this study
HZ-13	Topo-P3-TEM-R244S	derivative of XL1-blue	this study
HD-10	Topo-P3-TEM-T265M	derivative of XL1-blue	this study
HD-11	Topo-P3-TEM-R275A	derivative of XL1-blue	this study
JH-10	Topo-P3-TEM-R275L	derivative of XL1-blue	this study
JH-11	Topo-P3-TEM-R275Q	derivative of XL1-blue	this study
HZ-14	Topo-P3-TEM-N276D	derivative of XL1-blue	this study

HD-19	Topo-P3-TEM-L40V+I279T	derivative of XL1-blue	this study
HD-15	Topo-P3-TEM-D179E+M182T	derivative of XL1-blue	this study
HD-16	Topo-P3-TEM-E104K+M182T	derivative of XL1-blue	this study
HD-18	Topo-P3-TEM-R164C+M182T	derivative of XL1-blue	this study
HD-20	Topo-P3-TEM-R244H+L221M	derivative of XL1-blue	this study
HD-23	Topo-P3-TEM-G238S+T265M	derivative of XL1-blue	this study
HD-24	Topo-P3-TEM-G238S+R275L	derivative of XL1-blue	this study
HD-25	Topo-P3-TEM-M69L+R164H	derivative of XL1-blue	this study
HD-26	Topo-P3-TEM-M69L+R164S	derivative of XL1-blue	this study
HD-27	Topo-P3-TEM-M69V+R164H	derivative of XL1-blue	this study
LB01	Topo-P3-TEM-R164S+R244S	derivative of XL1-blue	this study
LB02	Topo-P3-TEM-R164S+N276D	derivative of XL1-blue	this study
LB03	Topo-P3-TEM-R164H+N276D	derivative of XL1-blue	this study
SM05	pBT-PaPb-TEM-1	derivative of XL1-blue	this study
SM06	pBT-P4-TEM-1	derivative of XL1-blue	this study
SM07	pBT-P5-TEM-1	derivative of XL1-blue	this study

Table S4. Antimicrobial susceptibility testing of the mutants generated in the E. coli XL1-blue background.

	Ampicillin	Amoxicillin	Piperacillin	Amx.(512)/ CLA	Amp.(512) /SUL	Pip.(512)/ TAZ	Amp(512)/ TAZ	Amp.(512)/ Avi	Ceftazidim	Ceftriaxon	Cefotaxim	Cefepim	Ceftobiprol	Aztreonam
XL1-blue	4	4	0.5						0.25	0.06	0.06	0.06	0.06	0.06
**SM-1 (TEM-1-pCR-Topo)**	**>1024**	**>1024**	**>1024**	**8**	**256**	**2**	**32**	**1**	**2**	**0.125**	**0.0625**	**1**	**2**	**0.5**
L40V	>1024	>512	>1024	1	16	1		2	0.125	0.03125	0.0625	0.0625	0.125	0.125
M69I	>1024	>1024	1024	32	64	2		1	0.25	0.03125	0.03125	0.03125	0.0625	0.0625
M69L	>1024	>1024	>1024	64	2048	16		2	0.25	0.0625	0.0625	0.25	0.5	0.25
M69V	>1024	>1024	>1024	32	128	4		1	0.25	0.03125	0.0625	0.125	0.25	0.0625
N100S	>1024	>1024	>1024	8	256	8		0.125	1	0.25	0.0625	1	2	1
E104K	>1024	>1024	>1024	4	256	4		1	8	1	0.25	2	4	2
S130G	>1024	>512	>1024	32	32	8		16	0.125	0.03125	0.0625	0.03125	0.25	0.125
S130T	64	256	32						0.125	0.03125	0.0625	0.03125	0.125	0.125
R164C	>1024	>1024	64	2	2		0.25	0.0625	8	0.0625	0.0625	0.5	0.25	0.125
R164H	>1024	>1024	1024	2	8	1		0.5	32	0.25	0.25	4	2	8
R164S	>1024	>1024	1024	2	2	0.25		0.5	64	1	0.5	8	2	8
W165C	>1024	>512	1024	4	256	1		1	1	0.0625	0.0625	0.25	0.5	0.125
W165G	>1024	>512	1024	8	64	1		1	4	0.0625	0.0625	0.125	0.5	0.125
W165L	>1024	>512	1024	16	256	1		2	2	0.03125	0.0625	0.25	0.5	0.25
W165R	>1024	>512	1024	8	128	1		2	2	0.0625	0.0625	0.25	0.5	0.25
M182T	>1024	>512	>1024	8	512	2		0.5	2	0.125	0.125	2	4	0.5
G238R	>1024	>512	>1024	8	256	2		0.5	0.5	0.125	0.0625	1	2	1
G238S	>1024	>1024	512	0.25	2		1	0.125	2	1	2	2	4	0.5
E240G	>1024	>1024	>1024	2	128	2		1	4	1	0.5	8	16	4
E240K	>1024	>512	>1024	4	512	16		1	4	0.25	0.125	1	1	1
E240R	>1024	>512	>1024	4	256	8		1	8	0.25	0.125	1	1	1
E240V	>1024	>512	>1024	0.5	8	1		0.5	2	0.125	0.0625	0.25	1	0.5
R244C	>1024	>512	>1024	32	32	1		2	0.125	0.03125	0.0625	0.0625	0.0625	0.125
R244G	>1024	>512	>1024	32	32	1		2	0.25	0.03125	0.0625	0.125	0.125	0.125
R244H	>1024	>512	>1024	32	32	1		2	0.25	0.03125	0.0625	0.03125	0.125	0.0625
R244L	>1024	>512	>1024	32	32	1		1	0.125	0.03125	0.0625	0.03125	0.0625	0.125
R244S	>1024	>1024	>1024	32	128	1		4	0.25	0.03125	0.03125	0.125	0.0625	0.0625

T265M	>1024	>512	>1024	8	1024	32		0.5	1	0.25	0.125	1	2	0.5
R275A	>1024	>512	>1024	16	256	2		1	1	0.0625	0.0625	1	1	0.125
R275L	>1024	>512	>1024	16	512	16		2	1	0.125	0.0625	2	2	0.25
R275Q	>1024	>512	>1024	16	512	8		2	1	0.0625	0.0625	2	2	0.125
N276D	>1024	>1024	>1024	32	1024	32		2	1	0.03125	0.03125	1	1	0.125
L40V+I279T	>1024	>1024	256	0.5	2		1	0.0625	0.25	0.03125	0.0625	0.0625	0.125	0.0625
D179E+M182T	>1024	1024	128	0.5	2		1	0.0625	64	1	0.5	4	4	0.5
E104K+M182T	>1024	>1024	>1024	4	256	0.5		0.5	16	1	0.5	2	4	2
R164C+M182T	>1024	>1024	1024	0.5	2	0.125		0.25	64	2	0.5	16	8	2
R244H+L221M	>1024	>1024	>1024	4	2	0.125		0.5	0.125	0.03125	0.0625	0.03125	0.0625	0.0625
G238S+T265M	>1024	>1024	1024	0.5	2	0.125		0.25	2	16	8	4	8	1
G238S+R275L	>1024	>1024	>1024	0.5	4	0.125		0.25	4	8	4	8	16	1
M69L+R164H	>1024	>1024	>1024	8	16	0.125		1	16	0.25	0.25	2	0.5	0.5
M69L+R164S	>1024	>1024	512	2	8		1	0.5	32	0.5	0.5	8	0.5	1
M69V+R164H	>1024	>1024	256	1	2		1	0.25	2	0.0625	0.0625	0.25	0.125	0.125
R164S-R244S	>1024	>512	512	16	8		4	2	1	0.0625	0.0625	2	0.125	0.5
R164S-N276D	>1024	>512	512	2	2		2	0.125	128	0.25	0.125	16	1	2
R164H-N276D	>1024	>512	512	2	2		4	0.125	32	0.125	0.125	4	1	1
**SM-2 (TEM-1-pBT)**	**>1024**	**>512**	**>1024**	**2**	**128**	**0.25**		**0.5**	**1**	**0.125**	**0.125**	**1**	**0.5**	**0.25**
Pa/Pb	>1024	>512	>1024	8	256	16		1	1	0.125	0.0625	1	2	0.25
P4	>1024	>512	>1024	8	256	16		1	2	0.25	0.125	1	4	0.5
P5	>1024	>512	>1024	4	256	8		0.5	1	0.125	0.0625	1	2	0.25

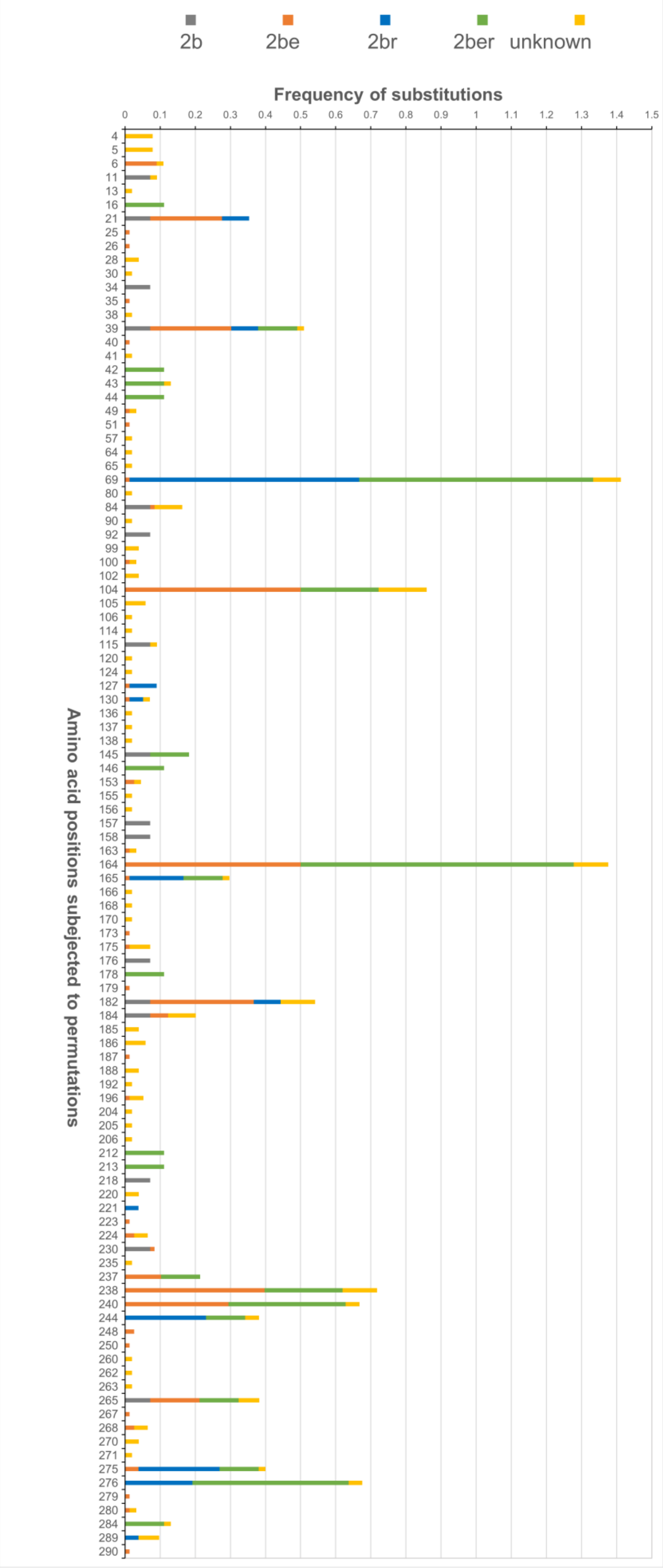

Figure S1. Frequency of amino acids substitutions in the TEM variants associated with a specific phenotype: 2b, 2be, 2br and 2ber, Mutational frequency in unknown variants are indicated as well. (Data as of 2017)

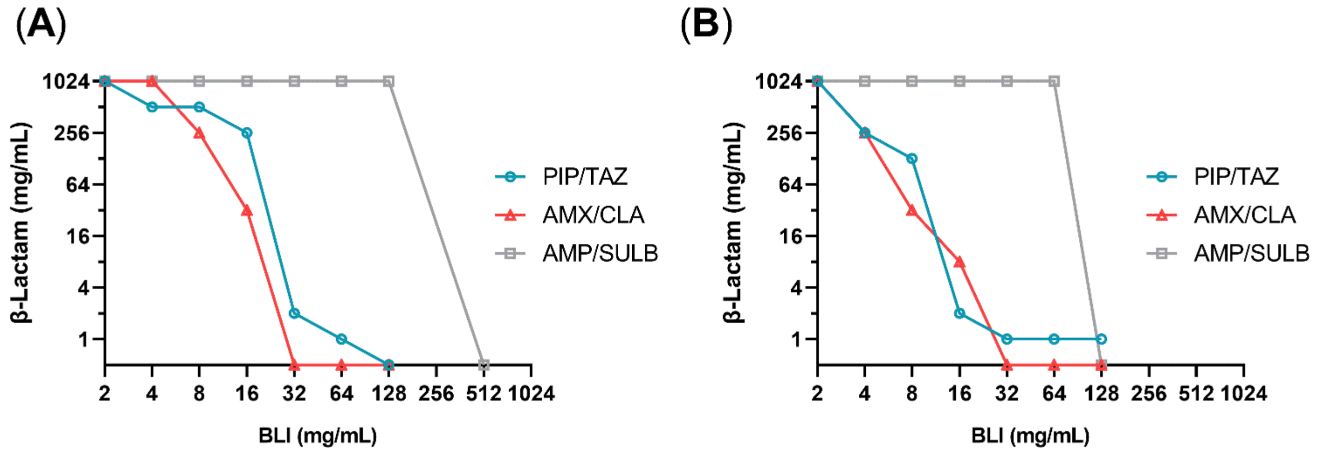

Figure S2. Isoboles of β-lactams combined with BLIs determined for (**A**) SM-1 and (**B**) SM-2. The curves represent the combined concentrations of the first nonturbid well in a checkerboard assay.

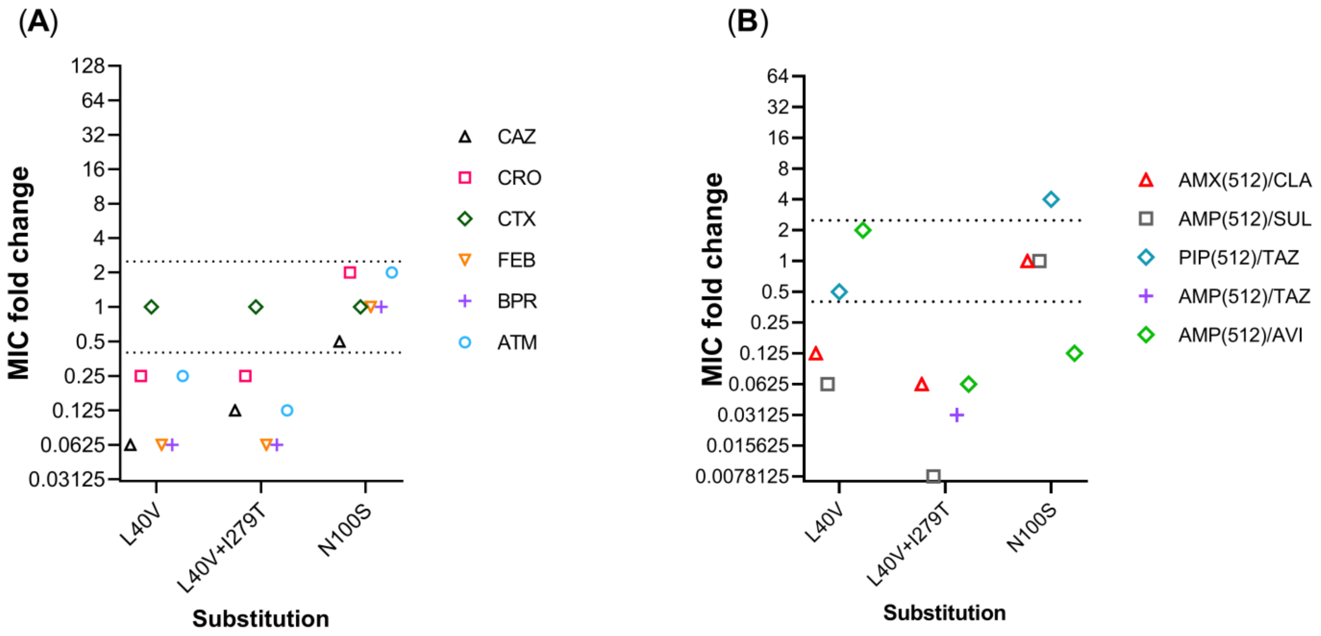

Figure S3. Changes in MIC of some "special cases" of mutations compared to the parenteral strain (SM-1) against (**A**) cephalosporins and aztreonam, or for (**B**) penicillin/BLI combinations.
